# Temporal turnover of the flora of lake islands: The island of Lake Pamvotis (Epirus, Greece)

**DOI:** 10.3897/BDJ.7.e37023

**Published:** 2019-08-21

**Authors:** Maria Sarika, Alexandros Papanikolaou, Artemios Yannitsaros, Theodoros Chitos, Maria Panitsa

**Affiliations:** 1 Institute of Systematic Botany, Section of Ecology and Systematics, Department of Biology, University of Athens, Athens, Greece Institute of Systematic Botany, Section of Ecology and Systematics, Department of Biology, University of Athens Athens Greece; 2 Division of Plant Biology, Department of Biology, University of Patras, Patras, Greece Division of Plant Biology, Department of Biology, University of Patras Patras Greece; 3 Goritsa Koutseliou Ioanninon, Ioannina, Greece Goritsa Koutseliou Ioanninon Ioannina Greece

**Keywords:** protected area, plant diversity, island diversity, turnover rate, monitoring, Lake of Ioannina

## Abstract

Lake Pamvotis is one of the Balkan "ancient" lakes, a Quaternary refugium of great environmental importance and ecological value, that is under various anthropogenic pressures. It belongs to a Natura 2000 Special Area for Conservation and Special Protection Area. Almost in the middle of the lake, there is an inhabited island - one of the two lake islands in Greece – that also attracts touristic interest. Τhe main objectives of the present study are to provide a floristic inventory of the protected island, combining data of two different sampling periods, within a 25 year interval, in order to estimate temporal beta diversity and species turnover of the island’s plant diversity. The value of the absolute and relative turnover rates of the floristic diversity of the island studied are 4.24 and 1.72, respectively and are amongst the higher rates reported for plants. The absolute difference between extinct (E) and immigrant (l) taxa is to a great extent accounted, concerning life forms, by therophytes (1.86), hemicryptophytes (1.56) and geophytes (1.04) and, for habitats, by taxa preferring agricultural and ruderal forms (2.52).

## Introduction

Lakes are considered as islands in many biogeographical and ecological respects and more specifically, they are “negative islands”, that is, they are more or less isolated freshwater areas surrounded by a hostile land matrix (Whittaker and Fernandez-Palacios 2007). Lake Pamvotis is one of the Balkan “ancient” lakes that are restricted within a radius of 300 km around Lakes Ohrid and Prespa (see Touka et al. 2018, Figure 1) and the term "ancient" refers to long-living modern or palaeo-lakes (Frogley et al. 2002; Frogley and Preece 2004). Lake Pamvotis has been in existence throughout the Plio-Pleistocene period, as shown by the identification of several endemic mollusc taxa which are known to be 500,000 years old and it has also been characterised as a Quaternary refugium (Frogley et al. 2001; Tzedakis et al. 2002).

**Figure 1.**
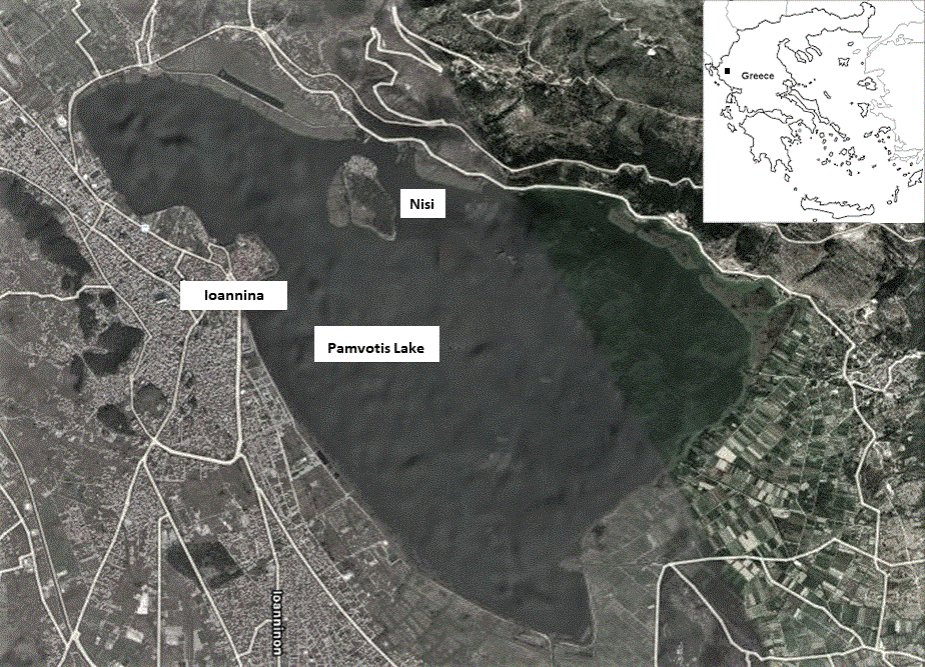
Map of the area studied.

The lake is located at the south-eastern foot of the mountain Mitsikeli and covers 22 km^2^ of the basin. It is a shallow lake with an average depth of 4.5 m (Sarika-Hatzinikolaou 1999; Sarika-Hatzinikolaou et al. 2003). It was formed by water concentration and is fed by the mountain Mitsikeli springs, while it has no physical surface outlet. The outflow of the water is carried through Lapsista’s ditch and is diverted to the river Kalamas. The lake belongs to the Natura 2000 Special Protection Area and Site of Community Importance GR2130005 and it is under the responsibility of the Lake Pamvotis Management Body. It has attracted research interests as a sedimentary archive on long term environmental and climate history and as a hotspot for European biodiversity (Touka et al. 2018). The lake could be characterised as an island (sensu Whittaker and Fernandez-Palacios 2007) and almost in the middle of the lake’s vast extent, opposite the town of Ioannina, a true island is formed, named “Nisi” (Fig. 1).

Chelli et al. (2019), demonstrated the urgent need for monitoring species diversity over time in protected areas by approaches that are scientifically sound and comparable over time and they underlined that the assessment of species diversity is crucial and that the analysis of temporal beta diversity – the variation of species composition over time - reflects species turnover. MacDonald et al. (2018) investigated relationships between fragmentation and species diversity concerning lake islands but there are very few previous studies on species turnover dealing with lake islands (e.g. Nilsson and Nilsson 1982).

In this context, the aquatic macrophyte flora of the Lake is well studied (Sarika-Hatzinikolaou 1999; Sarika-Hatzinikolaou et al. 2003; Stephanides and Papastergiadou 2007), but there is no previous literature information concerning temporal turnover of the plant species diversity of the island studied. Therefore, the changes that may have occurred over time in the floristic composition and plant species diversity on the island “Nisi” of Lake Pamvotis, which is heavily influenced by human interventions, have not yet been investigated. Τhe main objectives of the present study are to provide a floristic inventory of the protected island, by combining data of two different sampling periods in order to estimate the temporal beta diversity and species turnover of the island’s plant diversity within a 25-year period (1993-2019).

## Materials and Methods

### Survey area

The small island “Nisi” of Lake Pamvotis is a tectonic prominence of the alpine background of the area which is attributed to the same formation mechanism of the lake (Karakitsios 2005; Karakitsios 1995). The shape of the island is elongated, its relief is relatively mild, its highest elevation reaches 529 m above sea level or 59 m above lake level and its surface area is about 20 hectares. The island consists of limestone of the Upper Senonian (Upper Cretaceous). It is made up of compact strata of limestone with Rudist fragments (drifted from Gavrovo Zone) and layers of the pelagic limestone with Globotruncanidae. Their thickness vacillates between 200 to 400 m (Karakitsios 1995; Karakitsios 2005).

The area belongs to the wet bioclimatic level of the Emberger-Sauvage climatic diagram, with cool winters and the adverse xerothermic period for the plants is relatively short and not intense, lasting from the beginning of June until the middle of August.

The island has been inhabited since the 13^th^ century AD and its flora and vegetation have been strongly influenced by human activities. It is also an area of cultural interest attracting thousands of tourists every year.

### Data Collection, Database and Floristic Analysis

The present study is based on plant diversity data collected during two different sampling periods within a 25 year interval. During the first sampling period (sampling period A), Th. Chitos (TC) collected a large number of plant specimens by regularly visiting the island from autumn 1993 until autumn 1995. Α few taxa were also collected or observed by A. Yannitsaros, on a one-day visit to the island in October 1997 (Yannitsaros et al. 2005). During the second sampling period (sampling period Β) from May 2018 until April 2019, sample collections were carried out by Α. Papanikolaou and Μ. Panitsa. The sampling scheme, used for both sampling periods, included fieldwork (plant specimen collections and observations) all over the island during all seasons of each year.

Along with the terrestrial plants, aquatic plants growing close to island shores were also collected. Plant specimens are deposited in the personal herbaria of collectors TC and AY and in UPA Herbarium. The indication “obs.” means that the record is based on field observation.

For the determination of the plant material, Tutin et al. (1964), Tutin et al. (1981), Tutin et al. (1972), Tutin et al. (1976), Tutin et al. 1980, Strid and Tan (1997) and Strid and Tan (2002) were used. Taxonomy of the family of Poaceae follows Valdés and Scholz (2006). The families, genera, species and subspecies are listed in alphabetical order, within the major taxonomic groups. Nomenclature, life form, chorology, status categories and habitat preferences of plant taxa follow Dimopoulos et al. (2013). The main life form categories are summarised as follows: phanerophytes, chamaephytes, hemicryptophytes, geophytes, therophytes and aquatics. Chorological categories have been grouped into 5 main chorological elements: Widespread, Mediterranean, Balkan, Greek endemics and Alien.

In the framework of the authors' research, a list of all information recorded has been created including the plant taxa, their biological and chorological types and their habitat preferences, according to data provided by the “Vascular Plants of Greece" (Dimopoulos et al. 2013; see also "Flora of Greece web" 2017+, http://portal.cybertaxonomy.org/flora-greece-intro).

Absolute (**S2**; Herwitz et al. 1996) and relative (**Rt**; Diamond 1969, Morrison 2003) temporal turnover rates have been quantified using the formulas:

S2 = (I+E) / 2t

Rt = [(l+E) / t (SA+SB)] x 100

where **t** is the time period between censuses, **E** stands for species present only in sampling period A (extinct), **l** for species present only in sampling period B (immigrants), **SA** for all species recorded in sampling period A and **SB** for all species in sampling period B.

## Results

Field investigations on the lake island "Nisi" during two sampling periods, sampling period A (1993-1995, 1997) and sampling period B (2018-2019) revealed a total number of 350 plant taxa recorded, of which 5 are Pteridophyta, 3 Gymnospermae and 342 Angiospermae, belonging to 246 genera and 84 families, as is shown in Table 1. The total numbers of families and genera in sampling period A were 79 and 204, respectively, while in sampling period B, 71 and 163, respectively. A total of 263 taxa were present during sampling period A (SA) and 231 in sampling period B (SB). The total number of taxa present only in sampling period A was 119 (l) consisting 34% of the total flora, while 87 taxa (24.85%) were present only in sampling period B (E); 144 taxa (41.15%) were common to both sampling periods (C).

Asteraceae (46 taxa), Poaceae (34 taxa), Lamiaceae (26 taxa), Fabaceae (21 taxa), Brassicaceae (22 taxa) and Apiaceae (14 taxa) are the richest in taxa families in the flora of the island. Taxa belonging to these families represent 46.2% of the total island's flora. The richest in taxa genera are: *Trifolium* (8 taxa), *Veronica* (7 taxa), Medicago (5 taxa), *Bromus* (5 taxa), *Crepis* (4 taxa) and *Geranium* (4 taxa). The genus *Erigeron* is represented by 3 alien taxa. Most of the genera are represented by less than 3 taxa.

**Figure 2.**
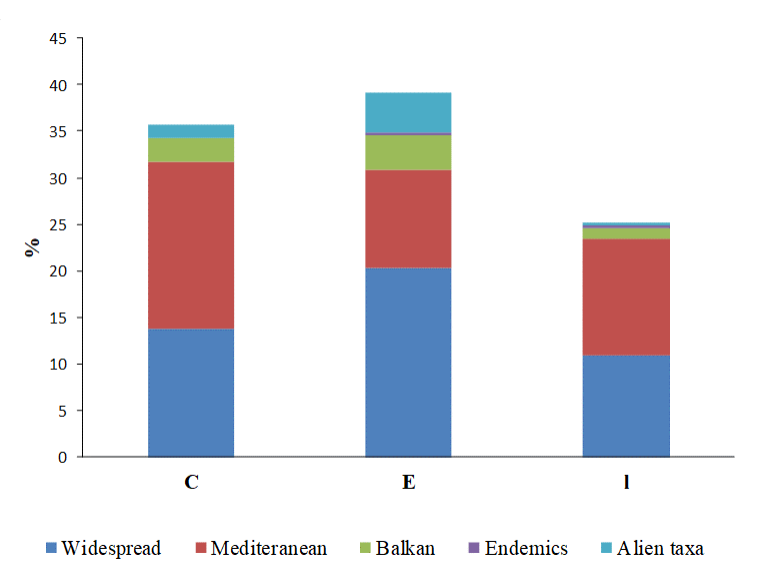
Proportions of chorological categories of the total plant taxa diversity including taxa recorded only in sampling period A (**E**), only in sampling period B (**l**) and taxa recorded in both periods (**C**).

Regarding the chorology, widespread taxa dominate (78.7%), followed by Mediterranean elements (33.1%) and alien taxa (6%). Fig. 2 presents proportions of different chorological elements recorded during the two sampling periods. The presence of Balkan elements is quite significant since 15 taxa (4.2%) have been registered, of which 6 are range-restricted (Table 1), while *Heliotropium halacsyi* Riedl is the only Greek endemic and range-restricted taxon registered. Interesting is the presence of widespread taxa that are rather rare in Greece like *Cicuta virosa*, a wetland toxic plant, which was found in a small population (not more than 1000 individuals) near the island shore, adapted to the lake's ecological conditions but at risk because of anthropogenic interference. It is classed as Vulnerable in Greece (Sarika and Yannitsaros 2009).

**Figure 3.**
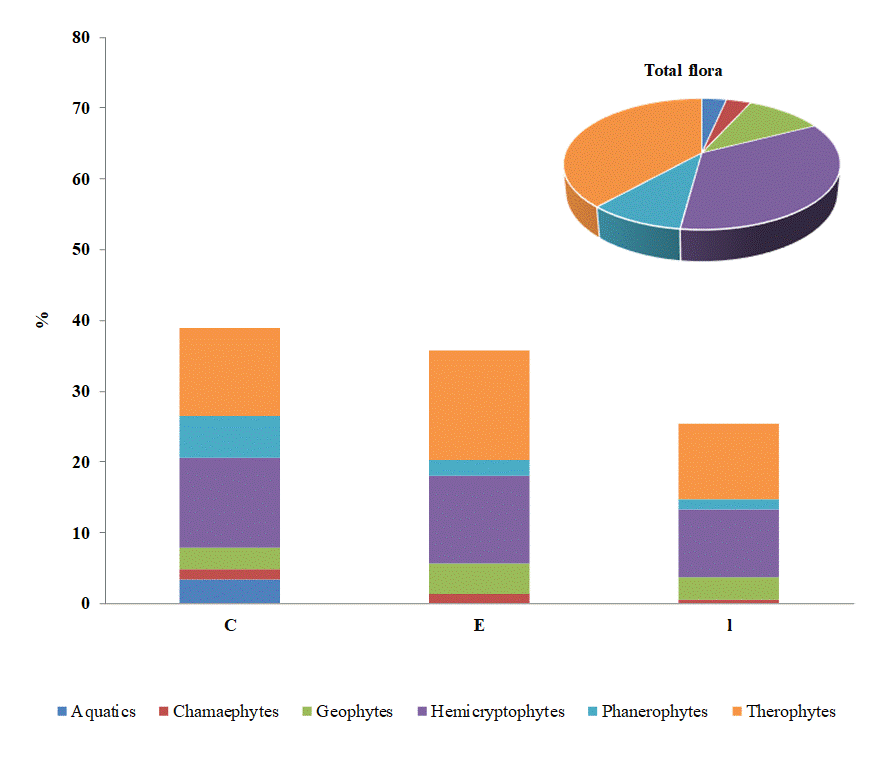
Life form spectrum of the total plant taxa diversity and proportions of different life forms for taxa recorded only in sampling period A (**E**), only in sampling period B (**l**) and taxa recorded in both periods (**C**).

**Figure 4.**
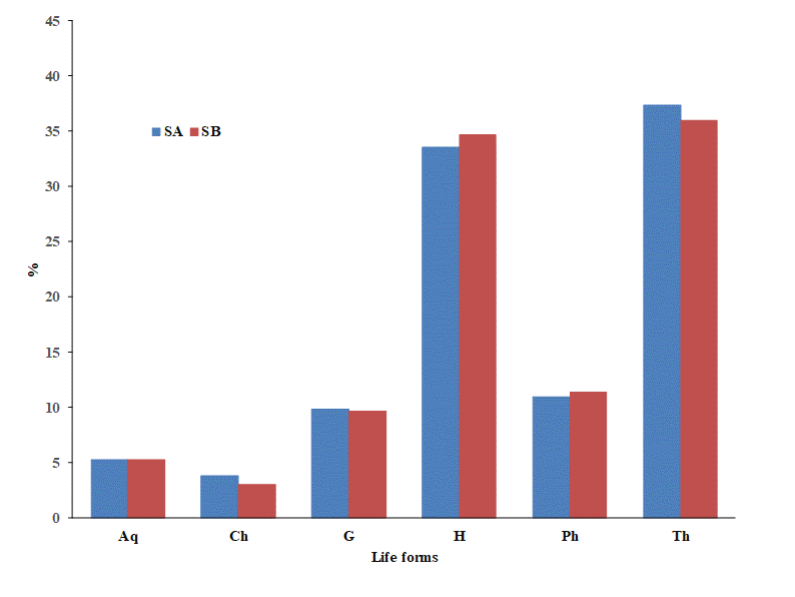
Percentage representation of life forms of plant taxa recorded in sampling periods A (**SA**) and B (**SB**). Abbreviations of life forms: phanerophytes (**Ph**), chamaephytes (**Ch**), hemicryptophytes (**H**), geophytes (**G**), therophytes (**Th**) and aquatics (**Aq**).

On the life form spectrum, Therophytes dominate (38.5%) followed by Hemicryptophytes (34.8%) and Geophytes (10.7%). Life forms of taxa present only in sampling period A, only in sampling period B and common to both sampling periods are presented in Fig. 3 and proportions of different life forms recorded during the two sampling periods in Fig. 4. Phanerophytes present a rather low proportion and, amongst them, there are tree species like *Cupressus sempervirens* L., as well as cultivated stands of *Pinus brutia* which form artificial small woods in the study area.

**Figure 5.**
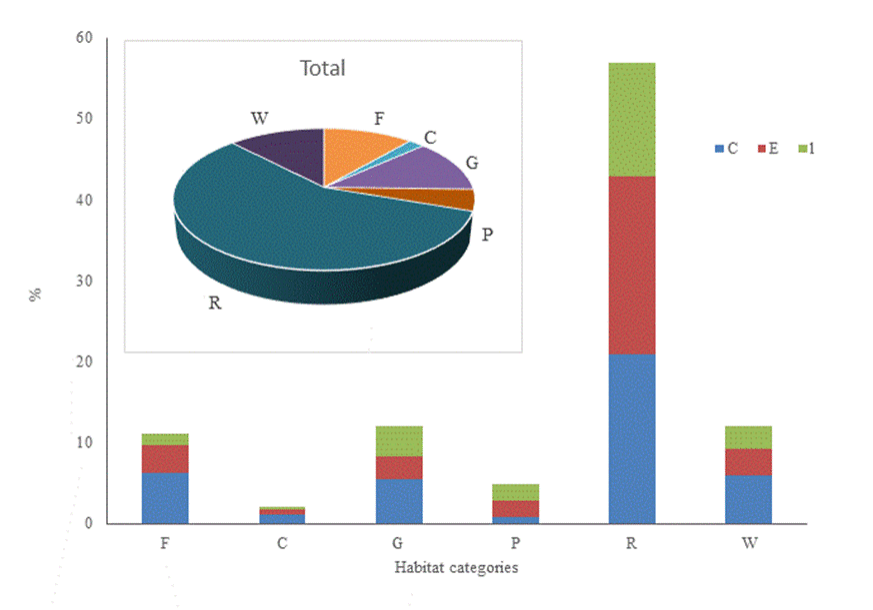
Proportions of different habitat categories for taxa recorded only in sampling period A (**E**), only in sampling period B (**l**) and taxa recorded in both sampling periods (**C**) and habitat preferences of the total plant taxa diversity: Freshwater habitats (**F**), Cliffs and rocks (**C**), temperate and submediterranean grasslands (**G**), xeric Mediterranean phrygana (**P**), agricultural and ruderal habitats (**R**) and Woodlands and scrub (**W**).

The evaluation on the habitat preferences of plant taxa reveals that most common are plants of agricultural and ruderal habitats (36.7%), followed by plants of grasslands (15.7%), of shrub formations (13.8%), of woodland and scrub (11.4%) and of freshwater habitats (11.1%) (Fig. 5).

Absolute turnover (S2) and relative turnover (Rt) rates for the total flora, for different life forms and for taxa with different preferences on habitats, are presented in Table 2. For the total flora, S2 is 4.24 species per year and Rt is 1.72 species per year (for t = 25 this gives 43% species change). The highest relative turnover rates amongst taxa of different life forms have been revealed for therophytes (2.05) and geophytes (2.16), while the highest absolute turnover rate concerning habitat preferences was calculated for taxa preferring agricultural and ruderal habitats (2.52). Relative turnover seems to be high for taxa preferring xeric phryganic formations because the percentage of these taxa in total is rather low (4.8%).

## Discussion

Lake Pamvotis is the second oldest lake at European level after Lake Ohrid and it is a sedimentary archive on long term environmental and climate history (Touka et al. 2018). Lake Pamvotis has also been characterised as a Quaternary refugium, that is an ecologically stable area, critical not only for the long-term survival of existing species, but also for the emergence of new ones (Tzedakis et al. 2002). Lake Pamvotis island is one of the two lake islands occurring in Greece - the second one is the island of Agios Achilleios in Prespa Lake - both belonging to protected areas. There has been a gap so far concerning plant species richness and temporal turnover of the diversity of the island studied, which is included in the protected area of Lake Pamvotis, GR2130005.

During our field investigations on the island in two sampling periods with a 25 year interval, 351 plant taxa were registered in total belonging to 84 families and 246 genera. Of the taxa recorded in total, 42.4% belong to the families Asteraceae, Poaceae, Lamiaceae, Fabaceae and Brassicaceae, which are amongst the best-adapted families to the ecological conditions of the Mediterranean area, as is confirmed by many floristic studies of Greek insular areas (amongst others Panitsa and Tzanoudakis 2001; Panitsa and Tzanoudakis 2010; Kougioumoutzis et al. 2012; Iliadou et al. 2014a; Iliadou et al. 2014b). This is also the case when floristic composition of the two sampling periods (SA and SB) is examined separately (Table 1).

The high percentage of Mediterranean taxa (41.1% in total, 28.6% in SA & 30.6% in SB) in conjunction with the high percentage of therophytes (38.3.1% in total, 37.3% in SA & 35.9% in SB) reflect the Mediterranean character of the flora of the Lake Pamvotis island. The island’s habitats types are not significantly at risk by the invasion of alien taxa since the percentage of alien taxa is about 6%, close to the one recorded for the Greek flora as a whole (5% according to Arianoutsou et al. 2010; 3.8% according to Dimopoulos et al. 2013), but in a small area. Dimitrakopoulos et al. (2017) recognised i) the human population density as a significant predictor of the spatial distribution of alien species in Greek Natura 2000 sites, assuming a positive relationship between human presence and alien plant species richness and ii) a possible preference of invasive plants for northern-western Greek Natura 2000 sites that could be explained by the cross-border trade that may facilitate the spread of invasive species.

The Pamvotis island plant diversity lost 10.5% of plant taxa during the studied interval of 25 years. The patterns of occurrence have changed for 60.4% of the plant taxa with 43% having being subjected to maximum turnover. It has to be noted that the absolute difference between extinct (E) and immigrant (l) taxa is mainly accounted by therophytes, hemicryptophytes and geophytes.

The value of turnover rate on the studied island is amongst the largest reported for plants (amongst others Cody 2006; Panitsa et al. 2008) and two factors should be taken into account, the small surface area (20 ha) and the continental character of the island. Cody (2006) showed that plant species turnover is a highly variable phenomenon, depending on the functional traits of each species, the local geomorphology and the ecological conditions of the islands. Morrison (2016) and Chelli et al. 2019) mentioned that temporal analyses could be affected by the “pseudoturnover” effect, the imperfect detection of species because of, for example, their presence during the sampling period only in the soil seed or bulb banks, but this is, more or less, the case for all plant species turnover studies. In order to avoid this "pseudoturnover" effect, the adequate time framework for the study of temporal plant species turnover on islands must include fieldwork distributed over all the different seasons per year and exhaustive field collections and observations all over the islands. This is what has been followed for the studied island.

Human activities are amongst the factors enhancing turnover (Panitsa et al. 2008). It is noteworthy that the absolute turnover rate also reveals the high contribution of ruderal plant taxa. Ruderal taxa are typically occurring and prevailing in disturbed areas, in agricultural and ruderal habitats and especially in sites with pronounced direct or indirect human activity, rural and urban sites, roadsides, excessively grazed and trampled sites, as well as naturally nutrient-rich and frequently disturbed pioneer habitats (Dimopoulos et al. 2013). Pyšek et al. (2017) noticed that ruderal flora and vegetation generally represent one of the most dynamic floristic-vegetation complexes that have a huge impact on the homogenisation of biodiversity and vegetation. Dornelas et al. (2014) and McGill et al. (2015) mentioned that changes in community composition (temporal beta diversity) and spatial structure (spatial beta diversity) need much more work to be quantified, but our focus should also include the quality and the composition of biodiversity.

Protected areas are the most widely applied policy tool for biodiversity conservation and there is now an increasing need to incorporate social impacts in decision-making processes by providing accurate estimations and developing ways to forecast their change in the future (Jones et al. 2015, Jones et al. 2017, Jones et al. 2018). Lazoglou and Vagiona (2018) assessed the willingness of inhabitants of the nearby city of Ioannina to pay for environmental actions and projects for the management of the protected area of Lake Pamvotis. The lake, despite its environmental importance and ecological value, is under various anthropogenic pressures (Latinopoulos et al. 2018) and Lazoglou and Vagiona (2018) suggested that economic valuation could provide an effective tool for planning processes and decision-making policies for the protection of the environment. Chelli et al. (2019) demonstrated the usefulness of long-term plant diversity programmes for the study of species richness and patterns of species assemblages over time in a protected area. Social impacts of protected areas should not be seen as static concepts, but should be seen as dynamic and long-term factors which need to be incorporated in decision-making processes (Jones et al. 2017). The knowledge of the plant species diversity, the high turnover rate and the continuing human interference on the protected lake island studied should be considered for biodiversity conservation actions which should also concern the whole protected area of Lake Pamvotis.

## Figures and Tables

**Table 1.** List of plant taxa recorded. Abbreviations: Sampling period **A** = Collections and observations from fieldwork 1993-1995,1997, Sampling period **Β** = Collections and observations from fieldwork period 2018-2019, **X** = alien, xenophyte, non-native plant taxa including cultigens, permanently established in at least one floristic region of Greece and **r** = range-restricted plant taxon characterised by a restricted distribution, by populations occurring along a linear distance not exceeding 500 km.

**Plant Families**	**Plant taxa**	**A**	**B**	**Status**
**Pteridophytes**				
** Aspleniaceae **	*Asplenium ceterach* L.	+	+	
	Asplenium trichomanes L. subsp. trichomanes	+	+	
** Polypodiaceae **	Polypodium cambricum L. subsp. cambricum	+	+	
** Salviniaceae **	*Azolla filiculoides* Lam.	+	+	X
** Selaginellaceae **	*Selaginella denticulata* (L.) Spring		+	
**Gymnosperms**				
** Cupressaceae **	*Cupressus sempervirens* L.	+	+	
** Ephedraceae **	*Ephedra foeminea* Forskål	+	+	
** Pinaceae **	*Pinus brutia* Ten. (cultivated)	+	+	
**Angiosperms**				
** Acanthaceae **	*Acanthus spinosus* L.	+	+	
** Alismataceae **	*Alisma lanceolatum* With.	+	+	
** Alliaceae **	*Allium ampeloprasum* L.		+	
	*Allium chamaespathum* Boiss.	+		
** Amaranthaceae **	*Amaranthus deflexus* L.	+	+	X
	*Amaranthus hybridus* L.	+		X
** Amaryllidaceae **	*Narcissus tazetta* L.	+	+	
	Sternbergia lutea (L.) Ker-Gawler ex Sprengel subsp. sicula (Tineo ex Guss.) D. A. Webb	+		
** Anacardiaceae **	*Pistacia terebinthus* L.	+	+	
** Apiaceae **	*Bubon macedonicum* L.	+	+	
	*Cicuta virosa* L.	+	+	
	*Conium maculatum* L.	+	+	
	Daucus carota L. subsp. carota	+		
	*Eryngium campestre* L.	+	+	
	*Ferula communis* L.		+	
	*Foeniculum vulgare* Miller	+	+	
	*Malabaila aurea* (Sm.) Boiss.	+	+	
	*Oenanthe aquatica* (L.) Poiret	+	+	
	*Opopanax hispidus* (Friv.) Griseb.		+	
	*Scandix australis* L.		+	
	Scandix pecten-veneris L. subsp. pecten-veneris	+	+	
	*Tordylium officinale* L.	+	+	
	Torilis arvensis (Hudson) Link subsp. arvensis	+	+	
** Apocynaceae **	*Nerium oleander* L.	+	+	
	*Vinca major* L.		+	
** Araceae **	*Arisarum vulgare* O. Targ. Tozz.		+	
	Arum italicum Mill. subsp. italicum (obs.)		+	
	*Arum maculatum* L. (obs.)		+	
	*Biarum tenuifolium* (L.) Schott (obs.)		+	
** Araliaceae **	Hedera helix L. subsp. helix	+	+	
** Asparagaceae **	*Asparagus acutifolius* L.	+	+	
** Asphodelaceae **	*Asphodelus ramosus* L.	+	+	
** Asteraceae **	*Anthemis cotula* L.	+	+	
	*Artemisia vulgaris* L.	+	+	
	*Bellis perennis* L.	+	+	
	*Bidens tripartitus* L.	+		
	*Carduus pycnocephalus* L.	+	+	
	Carduus tmoleus Boiss. subsp. tmoleus	+		
	*Carlina corymbosa* L. subsp. c*orymbosa*	+	+	
	Carthamus lanatus L. subsp. lanatus	+		
	*Centaurea calcitrapa* L.	+		
	*Centaurea graeca* Griseb.	+	+	r
	Centaurea solstitialis L. subsp. solstitialis	+		
	*Chondrilla juncea* L.	+		
	*Cichorium intybus* L.	+	+	
	*Crepis dioscoridis* L.	+	+	
	*Crepis rubra* L.		+	
	*Crepis sancta* (L.) Bornm.	+		
	*Crepis setosa* Haller fil.	+		
	Echinops sphaerocephalus L. subsp. albidus (Boiss. & Spruner) Kožuharov	+		
	*Erigeron bonariensis* L.	+	+	X
	*Erigeron canadensis* L.	+		X
	*Erigeron sumatrensis* Retz.	+		X
	Eupatorium cannabinum L. subsp. cannabinum	+		
	*Filago germanica* (L.) Huds.	+		
	*Galactites tomentosus* Moench	+		
	*Helminthotheca echioides* (L.) Holub	+		
	*Lactuca serriola* L.	+		
	Lactuca viminea (L.) J. Presl & C. Presl subsp. viminea	+		
	Leontodon crispus Vill. subsp. crispus	+		
	Leontodon hispidus L. subsp. hispidus		+	
	*Matricaria recutita* L.		+	
	Onopordum illyricum L. subsp. illyricum	+		
	Onopordum acanthium L. subsp. acanthium	+		
	*Pallenis spinosa* (L.) Cass.		+	
	*Pulicaria dysenterica* (L.) Bernh.	+	+	
	*Scolymus hispanicus* L.	+		
	*Senecio vernalis* Waldst. & Kit.	+	+	
	*Senecio vulgaris* L.		+	
	*Silybum marianum* (L.) Gaertn.		+	
	Sonchus asper (L.) Hill subsp. asper	+	+	
	*Sonchus oleraceus* L.		+	
	*Tanacetum parthenium* (L.) Sch. Bip.	+	+	
	Taraxacum sect. Fontana Soest		+	
	*Taraxacum* sp. (obs.)	+		
	*Urospermum picroides* (L.) Scop.	+	+	
	Xanthium orientale L. subsp. italicum (Moretti) Greuter	+		X
	*Xanthium spinosum* L.	+		X
** Boraginaceae **	*Anchusa officinalis* L.	+	+	
	*Anchusella cretica* Miller	+	+	
	*Cynoglossum columnae* Ten	+	+	
	Cynoglossum officinale L. subsp. officinale		+	
	*Echium italicum* L.	+	+	
	*Echium vulgare* L.	+	+	
	*Heliotropium europaeum* L.	+	+	
	*Heliotropium halacsyi* Riedl	+		r
	*Myosotis ramosissima* Rochel	+	+	
	*Symphytum bulbosum* K.F. Schimp.		+	
** Brassicaceae **	Aethionema saxatile (L.) R. Br. subsp. graecum (Boiss. & Spruner) Hayek	+		
	*Alliaria petiolata* (M. Bieb.) Cavara & Grande		+	
	*Alyssum alyssoides* (L.) L.	+		
	*Alyssum chalcidicum* Janka		+	r
	*Alyssum simplex* Rudolphi	+		
	*Arabidopsis thaliana* (L.) Heynh.	+		
	*Arabis hirsuta* (L.) Scop		+	
	*Arabis verna* (L.) R.Br.	+		
	*Aubrieta deltoidea* (L.) DC.	+	+	
	Aurinia saxatilis (L.) Desv. subsp. orientalis (Ard.) T. R. Dudley	+		
	*Berteroa mutabilis* (Vent.) DC.	+		
	*Bunias erucago* L.		+	
	*Capsella bursa* - *pastoris* (L.) Medicus	+	+	
	*Cardamine hirsuta* L. C.	+	+	
	Clypeola jonthlaspi L. subsp. jonthlaspi	+		
	*Draba muralis* L.	+	+	
	*Draba verna* L.	+	+	
	*Lepidium graminifolium* L.	+		
	*Lunaria annua* L. s.l.	+	+	
	*Rorippa amphibia* (L.) Besser	+		
	*Sisymbrium officinale* (L.) Scop.	+		
	*Thlaspi perfoliatum* L.	+		
** Butomaceae **	*Butomus umbellatus* L.	+	+	
** Caesalpinaceae **	*Cercis siliquastrum* L.	+	+	
** Campanulaceae **	Campanula sparsa Friv. subsp. sphaerothrix (Griseb)	+		
	*Campanula versicolor* Andrews (obs.)		+	
	*Campanula* sp.	+	+	
	*Legousia speculum*-*veneris* (L.) Chaix	+	+	
** Caprifoliaceae **	*Sambucus ebulus* L.	+	+	
	*Sambucus nigra* L.	+	+	
** Caryophyllaceae **	Cerastium brachypetalum Pers. subsp. roeseri (Boiss & Heldr.) Nyman	+		
	*Cerastium glomeratum* Thuill.	+	+	
	Petrorhagia illyrica (Ard.) P.W. Ball & Heywood subsp. haynaldiana (F.N. Williams) P.W. Ball & Heywood	+		
	*Petrorhagia obcordata* (Margot & Reuter) Greuter & Burdet	+		
	*Silene latifolia* Poiret	+	+	
	*Silene ungeri* Fenzl	+	+	r
	*Silene vulgaris* (Moench) Garcke		+	
	*Stellaria apetala* Ucria	+	+	
** Chenopodiaceae **	*Chenopodium giganteum* D. Don	+		X
	*Chenopodium album* L. (obs.)	+		
	*Chenopodium opulifolium* Schrader ex W.D.J. Koch & Ziz	+		
	*Dysphania ambrosioides* L.	+		X
** Convolvulaceae **	Calystegia sepium (L.) R. Br. subsp. sepium	+	+	
	*Convolvulus arvensis* L.		+	
	*Cuscuta campestris* Yunker	+	+	X
** Crassulaceae **	*Sedum acre* L.	+	+	
	*Sedum hispanicum* L.	+	+	
	*Sedum rubens* L.	+	+	
	*Umbilicus Umbilicus rupestris* (Salisb.) Dandy	+	+	
** Cucurbitaceae **	*Bryonia cretica* L.	+	+	
	*Ecballium elaterium* (L.) A. Richard	+		
** Cyperaceae **	*Carex divisa* Huds.		+	
	*Carex divulsa* Stokes		+	
	*Carex elata* All.		+	
	*Cyperus longus* L.	+	+	
	*Scirpus holoschoenus* L.	+	+	
	Scirpus lacustris L. subsp. lacustris	+	+	
	Scirpus maritimus L. subsp. maritimus	+	+	
** Dioscoreaceae **	*Dioscorea communis* L.		+	
** Dipsacaceae **	*Scabiosa tenuis* Boiss.	+		?r
** Euphorbiaceae **	*Euphorbia helioscopia* L.		+	
	*Euphorbia myrsinites* L.		+	
	*Euphorbia rigida* M. B.	+	+	
	*Mercurialis annua* L.		+	
** Fabaceae **	*Coronilla scorpioides* (L.) W.D.J. Koch		+	
	*Galega officinalis* L.	+		
	*Glyccyrhiza glabra* L.		+	
	Hippocrepis emerus (L.) Lassen subsp. emeroides (Boiss. & Spruner) Lassen	+	+	
	*Lathyrus setifolius* L.		+	
	*Medicago arabica* (L.) Hudson	+		
	*Medicago minima* (L.) Bartal.	+		
	*Medicago orbicularis* (L.) Bartal.	+	+	
	*Medicago polymorpha* L.		+	
	*Medicago rigidula* (L.) All.	+		
	*Robinia pseudacacia* L.		+	X
	*Trifolium dubium* Sibth.		+	
	*Trifolium incarnatum* L.		+	
	*Trifolium nigrescens* Viv.		+	
	*Trifolium pallidum* Waldst. & Kit.	+	+	
	Trifolium repens L. subsp. repens	+	+	
	*Trifolium resupinatum* L.	+		
	*Trifolium stellatum* L. s.l.	+		
	*Trifolium vesiculosum* Savi	+		
	*Trigonella spicata* Sm.		+	
	Vicia villosa Roth subsp. microphylla (Dum.-Urv.) P. W. Ball	+	+	
** Fagaceae **	*Quercus coccifera* L.	+	+	
** Fumariaceae **	Fumaria officinalis L. subsp. officinalis	+		X
** Geraniaceae **	*Erodium laciniatum* (Cav) Willd.		+	
	*Geranium brutium* Gasp.	+		
	*Geranium lucidum* L.	+	+	
	*Geranium molle* L.		+	
	*Geranium rotundifolium* L.	+	+	
** Hyacinthaceae **	*Bellevalia hyacinthoides* (Bertol.) K. M. Perss. & Wendelbo	+		
	*Muscari neglectum* Guss. ex Ten.	+	+	
	*Ornithogalum montanum* Cyr.	+	+	
**Hydrocharidaceae**	*Hydrocharis morsus-ranae* L.	+	+	
** Hypericaceae **	*Hypericum perforatum* L.	+	+	
	*Hypericum spruneri* Boiss.	+	+	
** Iridaceae **	*Hermodactylus tuberosus* L. (obs.)	+		
	*Iris* sp.		+	
** Juglandaceae **	*Juglans regia* L.	+		
** Juncaceae **	*Juncus inflexus* L.	+	+	
** Lamiaceae **	Ajuga chamaepitys (L.) Schreb. subsp. chia (Schreb.) Arcang.		+	
	*Ballota nigra* L.	+		
	Calamintha nepeta (L.) Savi subsp. glandulosa (Req.) P. W. Ball	+		
	*Lamium amplexicaule* L.	+	+	
	*Lamium bifidum* Cirillo		+	
	Lamium garganicum L. subsp. laevigatum Arcangeli	+	+	
	*Lycopus europaeus* L. (obs.)	+	+	
	*Marrubium alternidens* Rech.	+		
	*Marrubium peregrinum* L.	+		
	*Melissa officinalis* L.	+	+	
	*Mentha aquatica* L.	+	+	
	*Mentha pulegium* L.	+	+	
	Mentha spicata L. subsp. condensata (Briq.) Greuter & Burdet		+	
	*Micromeria juliana* (L.) Bentham ex Reichenb.	+	+	
	*Origanum vulgare* L.		+	
	*Phlomis fruticosa* L.	+	+	
	*Prasium majus* L.		+	
	*Salvia pratensis* L.		+	
	*Salvia sclarea* L.		+	
	*Salvia fruticosa* Mill.		+	
	*Scutellaria rupestris* Boiss. & Heldr. in Boiss.	+		r
	Sideritis romana L. subsp. purpurea (Talbot ex Bentham) Heywood	+		
	*Stachys palustris* L.	+	+	
	*Stachys tymphaea* Hausskn.	+	+	
	*Teucrium capitatum* L.		+	
	*Teucrium scordium* L.		+	
** Lauraceae **	*Laurus nobilis* L	+	+	
** Lemnaceae **	*Spirodela polyrhiza* (L.) Schleiden	+	+	
** Lentibulariaceae **	*Utricularia vulgaris* L.	+	+	
** Lythraceae **	*Lythrum salicaria* L	+	+	
** Malvaceae **	*Abutilon theophrasti* Medicus	+		
	*Alcea setosa* (Boiss.) Alef.	+	+	
	*Malva sylvestris* L.	+	+	
** Moraceae **	*Ficus carica* L.	+	+	
** Nyctaginaceae **	*Mirabilis jalapa* L. (obs.) (cultivated for ornament and naturalized)	+		X
** Oxalidaceae **	*Oxalis corniculata* L.	+	+	
** Oleaceae **	*Olea europaea* L.	+	+	
** Orchidaceae **	*Ophrys helenae* Renz		+	r
	*Ophrys lutea* Cav.		+	
** Orobanchaceae **	*Bellardia trixago* (L.) All.		+	
	*Orobanche* sp.		+	
** Papaveraceae **	Papaver somniferum L. subsp. somniferum	+	+	
	*Papaver dubium* L. s.l.	+		
	*Papaver rhoeas* L. (obs.)		+	
** Phytolaccaceae **	*Phytolacca americana* L.	+		X
** Plantaginaceae **	*Plantago lanceolata* L.		+	
	Plantago major L. subsp. major	+	+	
** Platanaceae **	*Platanus orientalis* L. (cultivated)	+	+	
** Plumbaginaceae **	*Plumbago europaea* L. (obs.)	+		
** Poaceae **	*Paspalum distichum* L.	+		X
	*Agrostis stolonifera* L.	+		
	*Alopecurus aequalis* Sobol	+		
	*Arrhenatherum elatius* (L.) J. Presl & C. Presl		+	
	*Avena barbata* Link		+	
	Avena sterilis L. subsp. ludoviciana (Durieu) Gillet & Magne	+	+	
	*Briza maxima* L.	+	+	
	*Bromus diandrus* Roth		+	
	*Bromus hordeaceus* L.		+	
	Bromus japonicus Thunb. subsp. phrygius (Boiss.) Pénzes	+	+	
	*Bromus madritensis* (L.) Nevski	+		
	*Bromus sterilis* L.		+	
	*Catapodium rigidum* (L.) C. E. Hubb.	+	+	
	*Cynodon dactylon* (L.) Pers.	+		
	*Cynosurus echinatus* L.	+	+	
	*Dactylis glomerata* L. s.l.	+	+	
	*Dasypyrum villosum* (L.) P. Candargy	+	+	
	*Digitaria ciliaris* (Retz.) Koeler	+		X
	*Holcus lanatus* L.	+		
	*Hordeum bulbosum* L.		+	
	*Hordeum murimum* Hudson		+	
	*Lolium perenne* L.		+	
	*Melica transsilvanica* Schur	+	+	
	*Parvotrisetum myrianthum* (Bertol.) Chrtek	+		
	*Phalaroides arundinacea* (L.) Rauschert	+		
	*Phleum phleoides* (L.) H. Karst.		+	
	*Phleum pratense* L.		+	
	*Phragmites australis* (Cav.) Trin. ex Steud.	+	+	
	*Poa annua* L.	+	+	
	*Poa bulbosa* L. s. str.	+		
	*Poa trivialis* L.		+	
	*Rostraria cristata* (L.) Tzvelev	+	+	
	*Secale strictum* (C. Presl) C. Presl		+	
	*Setaria viridis* (L.) P. Beauv.	+		
** Polygonaceae **	*Fallopia convolvulus* (L.) A. Löve	+		
	*Persicaria amphibia* (L.) Gray	+	+	
	Persicaria lapathifolia (L.) S. F. Gray subsp. lapathifolia	+		
	*Polygonum arenarium* Waldst. & Kit.	+		
	*Rumex crispus* L.		+	
	*Rumex palustris* Sm.	+	+	
	*Rumex pulcher* L.	+	+	
	*Rumex* sp.	+		
** Portulacaceae **	*Portulaca oleracea* L. s.l. (obs.)	+		
** Potamogetonaceae **	*Potamogeton crispus* L.	+	+	
	*Potamogeton lucens* L.	+	+	
	*Potamogeton pectinatus* L.	+	+	
** Primulaceae **	*Anagallis arvensis* L.		+	
	*Asterolinon linum-stellatum* (L.) Duby	+	+	
	*Cyclamen hederifolium* Aiton	+	+	
** Ranunculaceae **	*Anemone pavonina* Lam.		+	
	*Clematis flammula* L.	+	+	
	Ficaria verna Huds. subsp. calthifolia (Rchb.) Nyman	+	+	
	*Nigella damascena* L.	+	+	
	*Ranunculus baudotii* Gaudr.	+	+	
** Rosaceae **	*Crataegus monogyna* Jacq.	+		
	*Potentilla reptans* L.		+	
	*Rubus sanctus* Schreb.	+	+	
	*Sanguisorba minor* Scop.		+	
** Rubiaceae **	*Galium aparine* L		+	
	*Galium elongatum* C. Presl	+		
	*Galium spurium* L.		+	
	*Sherardia arvensis* L.	+	+	
	*Theligonum cynocrambe* L	+		
** Ruscaceae **	*Ruscus aculeatus* L.	+		
** Saxifragaceae **	Saxifraga rotundifolia L. subsp. chrysospleniifolia (Boiss.) D. A. Webb.	+	+	
	*Saxifraga tridactylites* L.	+		
** Salicaceae **	*Populus alba* L.	+		
	*Populus х canescens* (Aiton) Sm.	+	+	
	*Salix alba L.*	+		
** Scrophulariaceae **	*Antirrhinum majus* L. (obs.)	+		X
	Kickxia elatine (L.) Dumort. subsp. crinita (Mabille) W. Greuter	+		
	*Scrophularia peregrina* L.	+	+	
	*Verbascum pulverulentum* Vill.	+		
	*Verbascum sinuatum* L.	+	+	
	*Verbascum undulatum* Lam.	+	+	
** Simaroubaceae **	*Ailanthus altissima* (Miller) Swingle	+	+	X
** Solanaceae **	*Lycium barbarum* L.	+		X
	*Hyoscyamus niger* L.	+		
	*Solanum dulcamara* L.	+	+	
	*Solanum nigrum* L.		+	
** Sparganiaceae **	*Sparganium erectum* L. s.l.	+	+	
** Typhaceae **	*Typha domingensis* (Pers.) Steudel	+	+	
** Ulmaceae **	*Celtis australis* L.	+	+	
	*Ulmus minor* Mill.	+	+	
** Urticaceae **	*Parietaria judaica* L.	+	+	
	*Parietaria lusitanica* L.		+	
	*Urtica dioica* L.	+		
	*Urtica pilulifera* L.		+	
	*Urtica urens* L.		+	
** Valerianaceae **	Centranthus ruber (L.) DC. subsp. sibthorpii (Heldr. & Sart. ex Boiss.) Hayek	+		
	*Valerianella discoidea* (L.) Loisel.		+	
	*Valerianella microcarpa* Loisel.	+		
	*Valerianella turgida* (Steven) Betcke	+		
** Verbenaceae **	*Verbena officinalis* L. (obs.)	+		
** Veronicaceae **	*Veronica anagallis-aquatica* L.	+	+	
	*Veronica arvensis* L.	+		
	*Veronica cymbalaria* Bodard	+	+	
	*Veronica glauca* Sm.	+		
	Veronica hederifolia L. subsp. hederifolia	+		
	*Veronica persica* Poiret	+	+	X
	*Veronica polita* Fries	+	+	
** Violaceae **	*Viola macedonica* Boiss. &Heldr.		+	
	*Viola odorata* L.	+	+	

**Table 2.** Absolute turnover (S2) and relative turnover (Rt) rates for the grand total, for different life forms and for taxa with different preferences on habitats.

		**S2**	**Rt**
Total flora	Plant taxa diversity	4.24	1.72
Life forms	Chamaephytes (Ch)	0.28	1.64
	Geophytes (G)	1.04	2.16
	Hemicryptophytes (H)	1.56	1.86
	Phanerophytes (P)	0.26	0.94
	Therophytes (Th)	1.86	2.05
Habitat preferences	Freshwater habitats (F)	0.34	1.11
	Cliffs and rocks (C)	0.06	1.09
	Temperate and submediterranean grasslands (G)	0.46	1.64
	Mediterranean phrygana (P)	0.28	2.8
	Agricultural and ruderal habitats (R)	2.52	1.85
	Woodlands and scrub (W)	0.42	1.33
